# Gender Dysphoria and Detransitioning in Adults: An Analysis of Nine Patients from a Gender Identity Clinic from Finland

**DOI:** 10.1007/s10508-025-03176-5

**Published:** 2025-05-20

**Authors:** Kaisa Kettula, Niina Puustinen, Lotta Tynkkynen, Liisa Lempinen, Katinka Tuisku

**Affiliations:** 1 Gender Identity Clinic, University of Helsinki, and Helsinki University Hospital, Helsinki, Finland; 2 Outpatient Clinic for Assessment of Ability to Work, University of Helsinki, and Helsinki University Hospital, PL 250, HUS, 00029 Helsinki, Finland; 3 The Doctoral Programme in Clinical Research, University of Helsinki, Helsinki, Finland

**Keywords:** Gender dysphoria, Detransition, Regret, Transgender, Gender identity disorder, ICD

## Abstract

The aim of this study was to analyze the pathways of detransitioning, which is a rare, but serious complication of gender-affirming treatments (GATs). The patient group consisted of all patients who were referred to the Helsinki University Hospital’s Gender Identity Clinic (GIC) wanting medical treatment for detransition from 2018 to 2019. A new assessment was made systematically and retrospective data were collected. The sample consisted of nine patients originally diagnosed with gender identity disorder (F64.0). Seven of them were assigned female at birth and two were assigned male at birth. All seven females at birth had “major” regret and both males at birth had “minor” regret. All patients except one male assigned at birth wanted their previous GAT to be reversed. The mean regret time (i.e., time from the first diagnosis of F64.0 to the beginning of the new evaluation period) was seven years. The detransitioners had a high number of psychiatric diagnoses. Childhood trauma, sexual abuse or rape, eating disorder symptoms, borderline personality, and psychotic symptoms were common among detransitioners. Retrospectively, the patients reported that the need for transitioning in the first place was not the transgender identity or gender dysphoria, but reasons related to the maturation process and unresolved psychological stressors. An assessment made by the psychologist at the GIC revealed childhood trauma and severe challenges in parenting and attachment. It is important to acknowledge, support, and evaluate those regretting treatments and/or detransition, and to learn from them.

## Introduction

Detransitioning is the act of reversing a gender transition and returning to live in one’s original gender role (Hall et al., 2021). While increasing numbers of adults and adolescents seek medical gender-affirming treatment (GAT) at gender identity clinics (GIC) in Western countries, the infrequent and rather unknown complication of detransitioning may become more frequent (Boyd et al., 2022; Hall et al., 2021; Littman, 2021). The medical challenges (irreversibility of genital surgery and some hormonal sequelae) urgently demand more research and protocols for detransitioning patients (Vandenbussche, 2021).

There is no established definition to assess regret associated with detransition. Based on Pfafflin’s (1993) regret classification, minor regret is a feeling of regret secondary to surgical complications or social problems, and major regret is a feeling of dysphoria secondary to the new appearance, or desires of pursuing a detransitioning surgery. Wiepjes et al. (2018) classified regret into three subtypes: social regret, true regret, and feeling non-binary. Narayan et al. (2021) proposed the following categorization: true gender-related regret, social regret, and medical regret. In addition, for some patients transitioning and detransitioning is an important part of their developmental trajectory, so they indicate no regret (Butler & Hutchinson, 2020).

In the first academic studies, regret and dissatisfaction to treatment are often used as synonyms and the studies are focused on regret after gender-affirmation surgery (GAS) (Blanchard et al., 1989; Lawrence, 2003; Lindemalm et al., 1986; Pfafflin, 1993). In this study, we decided to use Pfäfflin’s classification since it is the simplest definition in this question that lacks research.

The first academic studies that were not focused on only GAS were case studies and publications that showed concerns about the phenomenon (Butler & Hutchinson, 2020; Exposito-Campos, 2021; Guerra et al., 2020; Levine, 2018). The current research is still scant, and the performed studies are retrospective research (Guerra et al., 2020) with small study groups comparable with this study or research based on online questionnaires (Littman, 2021; Turban et al., 2021; Vandenbussche, 2021). Guidelines in transgender health should include both preventive strategies and treatment guidelines for occurrences of regret (Narayan et al., 2021).

The reasons for detransitioning among 237 online participants were: realizing gender dysphoria was related to other issues (70%), health concerns (62%), the transition did not relieve dysphoria (50%), or the participant found other ways to deal with dysphoria (45%), unhappy with the social changes (44%), and change in political views (43%) (Vandenbussche, 2021). A study with 100 detransitioners (Littman, 2021) who answered online questionnaires found the reason behind detransitioning for most participants (58%) was coming to the conclusion that their gender dysphoria was caused by trauma or a mental health condition. Among them, 29% detransitioned because of discrimination and external pressures and 16% had detransition experiences associated with a non-binary identification. Despite not being asked in the survey, 23% of the participants indicated that internalized homophobia and difficulty of accepting oneself as lesbian, gay, or bisexual were behind their gender dysphoria and their initial transition wishes. A surprisingly high percentage of the participants (37.4%) reported having been pressured to transition by, for example, clinicians, partners, friends, or society. Of those born female, 7.2% expressed misogyny. The detransition rate was 6.9% in a recent UK study (Hall et al., 2021) where 12 detransitioners were found among 175 patients in a GIC. An even higher percentage of detransitioners, 9.8% for transmen (Boyd et al., 2022), was reported among gender dysphoria patients in primary care in the UK.

In a widely cited systematic Swedish study (Dhejne et al., 2014), the regret rate was 2.2% (2.0% FtM [female to male] and 2.3% MtF [male to female]) when all the applications for legal gender and surgical sex reassignment were examined from 1960 to 2010. The mean regret time (i.e., the time from attaining a new legal gender to the regret application) was 7.5 years for FtM and 8.5 years for MtF. But when the most patients (between 2001 and 2010, 153 FtM and 260 MtF) applied for a new legal gender in 2001 and especially in 2006, the mean regret time would not have been met before the study ended in 2010. The regret rate was estimated at only 0.3% from 2001 to 2010. The conclusion of a decline of regrets over the studied period may have been premature.

There is an urgent need for guidelines for the clinical process of detransitioning (Exposito-Campos, 2021; MacKinnon et al., 2023). The detransitioners might need psychological and psychiatric help for coping with mental illnesses, gender dysphoria, and feelings of transition regret, as well as coping with transition-related changes and internalized homophobia. Medical help like hormone therapy and surgeries can be necessary. Needs for social help like hearing other detransitioning stories, linking patients to relevant supportive peer groups in person as well as online, and legal help like changing back to their legal gender marker and/or name have been identified (Butler & Hutchinson, 2020; Exposito-Campos, 2021; Vandenbussche, 2021).

The total number of referrals to GICs in Finland has increased 20-fold in the last 22 years (2007–2024), with the shift in sex ratio from a previous MtF majority to a clear FtM majority, following the international trend (Aitken et al., 2015; Bouman et al., 2016; Kaltiala-Heino et al., 2019; Wiepjes et al., 2018). The psychiatric assessment of both adolescents and adults seeking treatment for gender dysphoria has been centralized in Finland into two university hospitals, Helsinki University Hospital and Tampere University Hospital, and into youth and adult units. In both hospitals, the tasks of multidisciplinary teams are: differential diagnosis, clinical assessment of gender dysphoria, treatment planning, follow-up and coordination of GATs. The assessment period lasts approximately one year (Kaltiala-Heino et al., 2015) and comprises an initial assessment (by a nurse), a diagnostic assessment (by a psychiatrist or a psychiatrist-in-training using the Structured Clinical Interview for DSM-IV [SCID-4 and SCID-II] (First et al., 2005)), and a psychologist’s assessment. The publicly funded GAT includes hormone therapy, voice therapy and other phoniatric services, as well as facial hair removal, and chest reconstruction surgeries. Genital surgery was, according to Finnish legislation, exclusively available for transgender people with a diagnosis of Transsexualism (F64.0.) (Kettula et al., 2019).

The aim of this study was to critically examine the psychiatric evaluation process preceding GAT in Finland to decrease the occurrence of serious complications such as wishes to detransition. To achieve this goal, the characteristics and circumstances of patients seeking to detransition were examined systematically with an emphasis on the subjective implications of the transition and detransition process of these patients. Considering the significant increase in referrals and the shift in sex ratio, we believe the clientele of gender identity outpatient clinics is very different from what we saw in the clinics before the 2010s. More information is needed regarding this new clientele.

## Method

### Participants

This study was carried out in the (Adult) Gender Identity Clinic of Helsinki University Hospital (HUS). Approximately half of the adult patients referred to GIC in Finland end up in HUS GIC. The study participants consisted of all patients with detransitioning aims or ideas, whose new referral to the GIC was approved from January 2018 to December 2019 for a new clinical assessment. Before April 2023 in Finland (the new law Act on Legal Recognition of Gender 295/2023 came into force on April 3 in 2023), it was not possible to change the legal gender without an evaluation period in GIC. Publicly fund medical treatment for reversing transition, such as breast augmentation to correct previous breast masculinization, is accessible only via GIC in Finland. These 13 patients were identified from referrals to the clinic. The patient records were reviewed by a psychiatrist (N.P.) and a clinical psychologist (L.L.). Among the detransitioners, four patients were removed from the dataset: three for not originally coming to the GIC of Helsinki University Hospital but to Tampere University Hospital’s so-called second-opinion evaluation, and the fourth patient was diagnosed with psychosis after starting GAT, so in that case, it was not a question of a logically reasoned detransitioning and thereby was excluded. In total, nine patients were included in the dataset; seven (78%) were female-assigned at birth, and two (22%) male-assigned at birth. Sexual orientation (compared to gender at birth) was bisexual (5), asexual (1) or lesbian (1) for female-assigned at birth and heterosexual (1) or homosexual (1) for male-assigned at birth. In addition, two female-assigned at birth individuals identified as both asexual and lesbian or bisexual.

### Measures and Procedure

The initial assessment phase refers to the first assessment phase at the GIC (between the years 1996–2014), when patients were diagnosed with gender identity disorder (F64.0) and the detransition assessment phase to the second assessment period when patients were referred back to the clinic hoping to get medical treatment for detransition.

Baseline information was collected by a psychiatrist (K.K.) from previous patient records of Helsinki University Hospital and for three patients from previous patient records from Tampere University Hospital since the first diagnostic evaluation was done there. The patient records consisted of data entries from nurses, social workers, psychologists, and psychiatrists during the multi-professional assessment phase. In addition, the patient records included information, obtained with consent, on the patients’ previous psychiatric treatment. Psychiatric diagnoses obtained by structured interviews and patient records at the initial assessment period were compared to the psychiatric diagnoses at the recent assessment during detransitioning.

Information on the socioeconomic status, which includes the size of the household, marital status, and capacity to work/study, was recorded from the beginning and end of the initial assessment period and from the beginning of the detransition assessment phase. Information on psychiatric history, such as previous psychiatric hospitalizations, treatment in psychiatric outpatient unit and psychotherapy, was recorded. Additionally, suicide attempts and non-suicidal self-harm, as well as substance use, were recorded.

Details of the initial assessment period in the GIC were recorded (date of initiation, date of diagnosis, age at the time of initiation, sex assigned at birth, gender identity, sexual orientation, starting date of hormones, how hormones were started, date of any surgeries, all treatments for gender dysphoria, gender incongruity before puberty, the length of the initial assessment phase). Additionally, details of the detransition assessment phase were recorded (date of new initiation, age at the time of initiation, gender identity, date of hormones stopped, time from the first diagnosis to the new initiation, treatment needed).

During the detransition phase, all the patients were interviewed by a psychiatrist and diagnostically re-examined using the Structured Clinical Interview for DSM-IV (SCID-I and SCID-II) (First et al., 2005). Furthermore, eight were interviewed and assessed by a psychologist (L.L.) using clinical evaluation. For six patients, additional information was gathered using the Experiences in Close Relationships-Revised [ECR-R] self-report measure (Fraley et al., 2000). The ECR-R evaluates experiences related to adult relationships. It is based on the attachment style theory and the purpose of it is to evaluate adults’ romantic attachment. During the initial assessment period, the patient’s gender identity was defined by interview with open questions such as “How do you define your gender?” and with a detailed interview comprising the lifespan of the patient. In 2018, at the detransition assessment phase, the visual analog scales on experienced gender female (0–100), male (0–100), and other (0–100) were added to the evaluation (in addition to open questions and a lifespan interview), and the Gender Congruence and Life Satisfaction Scale (Bouman et al., 2016; Puustinen et al., 2024) on the experienced dysphoria was collected. Patients’ retrospective reflection and responses to the following study questions were collected from the patient data by a psychiatrist (K.K.): (1) Why did the patient want to transition in the beginning?; (2) How did they discover that transitioning was not the right choice?; (3) What could the GIC have done differently?; and (4) How did other people react to the patient’s wish to detransition?

## Results

During the initial assessment phase, all the patients’ identities were transgender. The diagnosis F64.0 was given at approximately the age of 25 (see Table 1 for mean, median, and standard deviation). The initial assessment phase lasted ca. 13 months. Three patients required a prolonged initial assessment phase due to the need for extra time to make sure the gender identity was stable enough, or due to urgent psychiatric care. The mean age at the beginning of detransition was 33 years. Five patients found their gender identity to align with their sex assigned at birth (two of them had returned twice to the GIC: during the first detransition assessment phase their identity was non-binary and at the second detransition assessment phase cis-gender). Three patients’ gender identity was non-binary and one was still transgender. The gender incongruence had originated in childhood (“early onset dysphoria”) with three patients wishing to detransition, and among six patients the incongruence first emerged during puberty (“late-onset dysphoria”).

**Table 1:** Patient and detransitioning characteristics

Results	Mean	SD	Median
Age (yr) diagnosis was given	25	11.7	21
Time (m) the initial assessment phase lasted	13	9,6	33
Age (yr) detransition assessment phase started	33	14	28
Regret time (yr)	7	4.8	6
Use of hormones (yr)	7	6.6	4

**Fig. 1:**
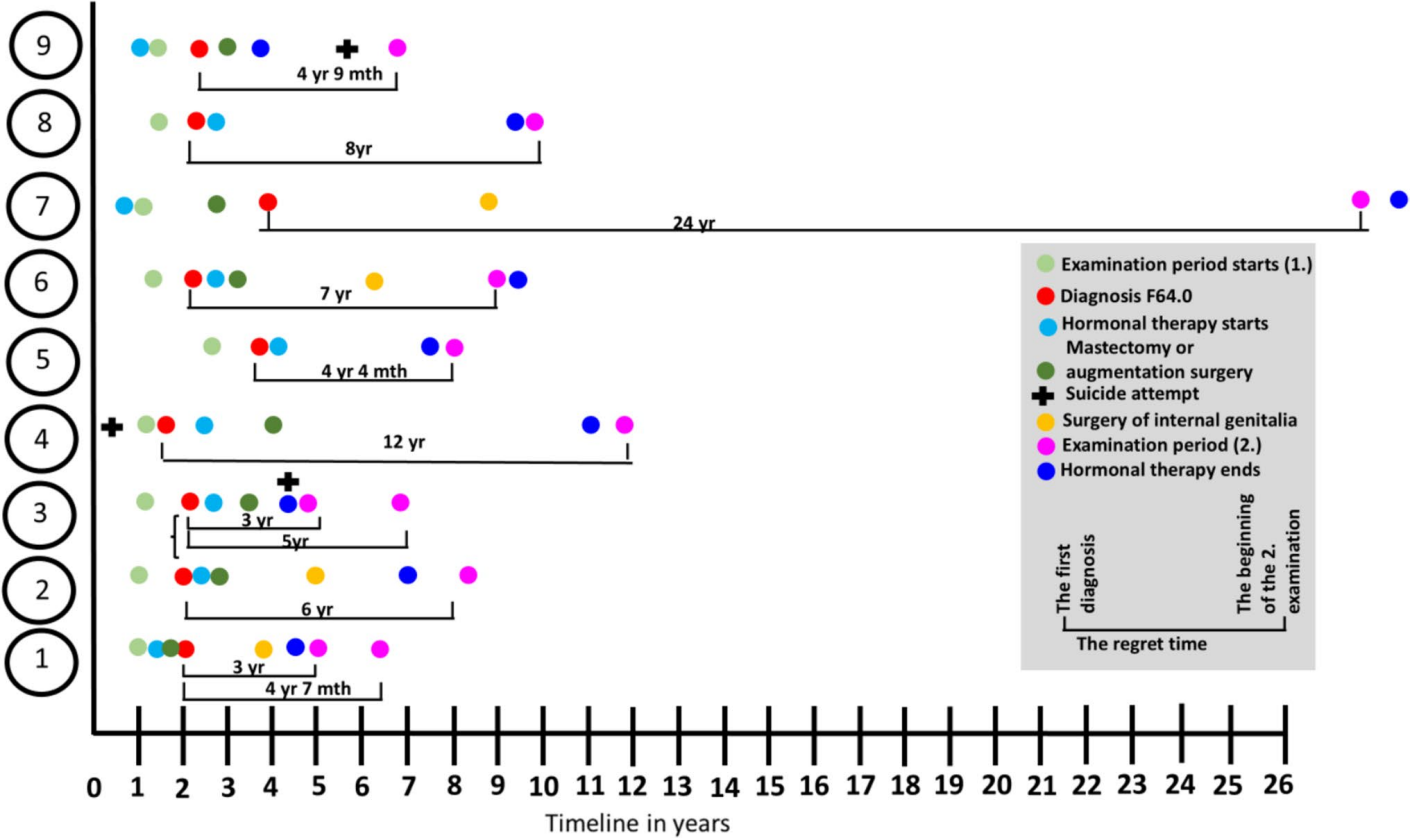
The patients’ initial and detransition assessment phases, suicide attempts, and treatments

The time from transition to regret was approximately seven years. Seven patients used hormones for approximately 4.3 years before deciding to stop. Two patients used hormones for much longer than the seven other patients, for nine and 23 years (Fig. 1). Three patients independently initiated hormonal therapy, and two patients had chest reconstruction surgery done in a private hospital before the initial psychiatric evaluation period was completed. In the end, eight patients had undergone chest reconstruction surgery or augmentation surgery. Two patients used epilation and phoniatric services. The female reproductive organs of four patients had been surgically ablated, but none had sex reassignment surgery. The patients’ assessment phase and treatments are visualized in Fig. 1. For detransition treatment, four patients needed permanent hormone replacement therapy, four chest reconstruction or augmentation surgeries, one phoniatric services, and five epilation. All except one wanted to correct their legal gender.

Patients’ ability to function was evaluated according to three different points of view: (1) their ability to work or study fulltime, (2) their ability to live alone, and (3) their ability to form a romantic relationship (Table 2). These aspects were chosen because they were accessible from the patient records. If the patients were not working or studying fulltime, they were on a sick leave or disability pension. Living independently means they did not live with their parents or in a care home. Being married or living with a partner was recognized as being in a relationship.

**Table 2:** Ability to function

Phases	Number of patients		
	Working/studying	Living independently	In a relationship
Beginning of the initial assessment phase	6	6	1
End of the initial assessment phase	4	8	3
Beginning of the detransition assessment phase	3	9	0

During the initial assessment phase at the HUS GIC, a psychiatric interview was performed for all patients seeking GAT. The records of these interviews for all the patients were reviewed by a psychiatrist (K.K.). Psychiatric diagnoses at the initial assessment period were compared to the psychiatric diagnoses at the recent assessment during detransitioning. The results are shown in Table 3. Only one of the patients had not had contact with the psychiatric outpatient unit. Three had been treated in the ward of a psychiatric hospital and six had or were in psychotherapy. Seven patients had a history of non-suicidal self-harm, and three patients had tried to commit suicide (one before the initial assessment phase, two after finishing the hormonal treatment and before starting the detransition assessment phase) (Fig. 1). Five had suffered from problems with substance abuse: three with alcohol, one with alcohol and cannabis, and one with amphetamine. All the patients had other psychiatric diagnoses in addition to gender identity disorder diagnoses (Table 3). For the detransition assessment phase, only two did not have additional psychiatric diagnoses. During the initial assessment phase, five patients were referred to psychiatric treatment (psychiatric outpatient unit or student health care). During the detransition assessment phase, seven patients were referred.

**Table 3:** Lifetime DSM Axis I and II diagnoses of detransitioners

Diagnoses	n_1_	n_2_
Mood disorders F30–39	9	2
*of which bipolar II (F31.8)*	2	0
Anxiety disorders (F40–48)	11	3
Personality disorders (F60)	3	3
*of which Borderline personality disorder (F60.3)*	2	3
Psychotic disorders (F20–29)	1	1
*reported symptoms*	4	0
Diagnoses due to psychoactive substance use (F10–19)	2	0
*reported problematic use*	4	0
Eating disorders (F50)	4	0
*reported symptoms*	3	0
Dissociative disorder (F44)	1	2
Neuropsychiatric disorders:		
Specific developmental disorders (F80–89) Behavioral disorders (F90–98)	1 0	1 1

n_1_ = All diagnoses at the initial assessment phase

n_2_ = New diagnoses at the detransition assessment phase

The most common psychiatric diagnoses among the patients were mood disorders (F30-F34): Two had bipolar II disorder and six had depression (changed to recurring depression for two patients at the detransition assessment phase). Anxiety disorders (F40–F48) were diagnosed in six of the patients. At the detransition assessment phase, one more patient was diagnosed with anxiety disorder and two had an additional anxiety disorder diagnosis. Personality disorder (F60) was diagnosed among three of the patients, of which there were two diagnoses of borderline personality disorder (F60.3). Three additional borderline personality disorders were diagnosed at the detransition assessment phase.

Patients often had several diagnoses from the same diagnostic main group (e.g., panic disorder and post-traumatic stress disorder from the anxiety disorders group). Two of the five patients who expressed psychotic symptoms were diagnosed with psychotic disorder (an organic psychosis with auditory hallucination and a transient psychotic disorder). The three additional patients expressing psychotic symptoms were later discovered to be related with no long-term schizophrenic disorders but borderline personality disorders and dissociative disorder. Four out of nine patients were diagnosed with eating disorders (F50–F59) with three additional having experienced symptoms of eating disorders (symptoms were not severe or frequent enough for a diagnosis). A neuropsychiatric diagnosis (F80–F89) was given at the initial assessment phase to only one patient, but by the time of the detransition phase, two more diagnoses (F80–F98) were set. Diagnosis for dissociative disorder (F44) was given at the initial assessment phase for one, and for two more during the detransition assessment phase.

### Findings from the Psychologist’s Assessment

According to the psychologist’s evaluation during the detransition assessment phase, none of the participants had a secure attachment style. Interviewing and observing social interaction were used as the psychologist’s evaluation methods. The parents were described as either weak and in need of protection or emotionally cold, violent, and abandoning. Because of the severe parental issues (physical neglect, physical and emotional abuse) and the insecure attachment style, all the patients were considered to have had a traumatic childhood. Among the six patients, whose attachment style was assessed with ECR-R, three were preoccupied, two avoidant, and one fearful.

During the initial assessment phase seven patients showed some concerns in the following areas: the discontinuity of identity, the fragility of self-esteem, immature coping mechanisms, inflexibility in the personality, and problems with reality testing. In addition to traumatic relationships with parents in childhood, other traumatic experiences were found: sexual abuse or rape (6 patients), loneliness or lack of friends (5), bullying at school (6), abusive mother (1) and psychological abuse in a romantic relationship (1). Seven patients regretted starting the evaluation process at the GIC and showed feelings of shame and guilt. One felt detransitioning was necessary for personal development, despite requiring medical treatment for detransitioning. One patient wanted to detransition partly because GAS was not possible due to medical reasons.

### Patients’ Retrospective Reflection and Responses to Study Questions

The patients’ subjective experiences were collected and classified from the patient records that consisted of data entries by nurses, social workers, psychologists, and psychiatrists by a psychiatrist (K.K.). The results are shown in Table 4.

**Table 4:** Patients’ retrospective reflection and responses to study questions

Reasons to transition in the beginning	Number of patients who mentioned it
Growing up as a woman was difficult	6
I had a feeling of “not belonging”	4
Being a trans man looked good/easier	4
The process gave structure and meaning to my life	3
I had bad experiences being a girl (e.g., sexual assault)	3
Appearance-oriented thinking/ I didn’t look good enough	3
It was more important not to be a woman than to be a man	2
I was afraid to grow up and become a woman	2
I did not like the attention I got as a girl	2
Lack of male/female role model growing up	2
I wanted to start my life over	1
I confused homosexuality with transsexuality	1
I do not know	1

Way of discovering that transitioning was not the right choice	Number of patients who mentioned it
I was more in touch with my feelings/body	3
I realized I was non-binary	3
I realized I had a dissociative disorder	2
Psychotherapy helped me	2
I realized that women can also be masculine	1
My self-esteem got better	1
The treatment did not impact my mood or looks	1
I did not “pass” as a woman/man	1
I did not get the treatments I wanted	1
I was in a stable and safe place in my life	1
I had a religious awakening	1
My chance for finding a partner is better	1

Wishes/feedback to the Gender Identity Clinic at the detransition assessment phase	Number of patients who mentioned it
“Examine me more” (e.g., trauma, dissociative disorder, autism)	3
Nothing	2
Direct me to psychotherapy	1
Get in touch with my psychiatrist	1
Make it easier to contact the GIC if regretting	1
Take time to discuss about gender and sexuality	1
Should not have tried to convince me I was trans	1
Should have told me I was not ready	1

Other people’s reactions to detransitioning	Number of patients who mentioned it
I got peer support	2
I lost some friends	2
I am worried what trans people might think	2
I am not welcome in trans communities	2
People have been hostile	2
Some friends started to think about detransitioning	1
My family was pleased	1

## Discussion

The number of detransitioners appears to be growing. Compared to a Swedish study in 2015 (Dhejne et al., 2011) where 15 people wanted to detransition during a 50-year period, in this study there were nine detransitioners in two years (2018–2019). The following year the number of detransitioners was even higher: nine in one year (2020). In addition, it is probable that this study or any study about detransitioning is likely to miss a fair number of detransitioners. Returning to the clinic is mentally burdensome for many reasons. Only the patients who seek treatment or are applying for a new legal gender will return to the GIC. In addition, they need to be in a psychiatric condition that allows them to do it. In a recent online study (Littman, 2021), only 24% of detransitioners informed their doctor or clinic that they had detransitioned. In another online study (Vandenbussche, 2021), only 29% of detransitioners reported getting help from the same doctor as in their transition. The regret rates today are probably higher than previously assumed. This study was not able to determine the regret rate because the naturalistic sample consisted of only those detransitioners who contacted our clinic on their own initiative.

The delay in detransitioning seems to be decreasing. The regret time in this study (seven years) and the Swedish study (eight years) (Dhejne et al., 2011) was similar. Two patients’ regret times and use of hormones were clearly longer than the others’ (Fig. 1) in this study. If only patients who were evaluated after 2010 are taken into consideration, the regret time is five years, and hormonal therapy was used for approximately 4.3 years. In Littman’s study (2021), the participants remained transitioned for approximately 3.9 years and in a similar study by Vandenbussche (2021) for 4.7 years.

Two patients had two detransition assessment phases: at first presenting as non-binary gender identity and later cis-gender identity. Non-binary gender identity is a possible stage for detransitioning as with transitioning, when moving on the continuum of gender identities. Surprisingly, not all detransitioners’ gender incongruence had emerged during puberty (“late-onset dysphoria”) (Kaltiala-Heino et al., 2015): For three, it had started in childhood. According to these data, an anamnesis of early onset gender dysphoria did not exclude later wishes to detransition. This may be explained by the traumatic childhoods of all the study participants in this sample.

Collecting data on detransitioning using the help of transgender advocacy groups risks leaving out politically inactive people (D'Angelo et al., 2021). The people reached by advocacy groups are likely to be predominantly people who are politically aligned and engaged in specific political actions of advocating for increased access to transition. The patients in this study reported that they avoided or had no contact with transgender advocacy groups, nor were they interested in going public with their experiences.

The patient group of this study is small, and it is difficult to generalize findings about patients’ diagnoses. Some conclusions can be drawn, however, to provide new guidelines for enhancing patient safety and avoiding adverse outcomes. According to Heylens et al.’s (2014) study (with similar percentages in Hall et al., 2021 and Boyd et al., 2022), a transgender individual’s lifetime prevalence of an axis I diagnosis was 70% (in this study of detransitioners, 100%). A transgender individual’s lifetime prevalence of affective problems according to Heylens et al. was 60% and anxiety disorders 28%, while in this study the prevalence was 89% for affective problems and anxiety disorders 78%. The axis II diagnoses were estimated at 15% for transgender individuals in Heylens et al.’s study and for detransitioners in this study 33%. Psychotic disorders were rare (1%) in Heylens et al.’s study, but 16 patients (13%) were excluded from the study because of clear psychotic symptoms at the time of application. In this study, 22% patients had a psychosis diagnosis. Although all psychiatric disorders were more common in detransitioners than transgender individuals according to Heylens et al., the borderline personality disorders, eating disorders and symptoms, and neuropsychiatric and psychotic disorders among them were especially high.

The reasons that patients had thought led to their gender dysphoria were undiagnosed dissociative disorders (33%) and trauma (only one patient was diagnosed with post-traumatic stress disorder [PTSD]). Sometimes patients and even the doctors referring patients to the GIC, consider an eating disorder as a sign of gender dysphoria (for trying to prevent secondary sex characteristics), but it is important to consider eating disorders in the differential diagnosis as conditions that are separate from the gender dysphoria. In this study, when comparing patients’ diagnoses at the assessment phase and at the detransition assessment phase, some differences can be found. Personality disorders were higher (change from 33 to 67%) at the detransition assessment phase. The prevalence of substance abuse disorders was 16% at the assessment phase but 56% at the detransition phase. Eating disorders were rare (2%) in the assessment phase but were more common in the detransition phase (44% with diagnoses, 78% had some symptoms).

Dhejne et al. (2011) found that even after GATs the risk of death by suicide, in-patient care for psychiatric disorders, and suicidal behavior is more common compared to matched controls. Seven patients in this study had a history of non-suicidal self-harm, and three patients had tried to commit suicide. It seems that the patients who end up wanting to detransition have a significant amount of psychiatric burden at their baseline before transition. This becomes evident in their ability to function; at the beginning of the initial assessment phase 67% of the patients could work or study and even fewer at the beginning of the detransition assessment phase (33%). This further confirms the need for a thorough psychiatric assessment as a part of the GIC services. When it was asked if the patients left psychiatric symptoms undisclosed during the initial assessment phase, only one patient confirmed doing so. This study does not support the hypothesis that patients feel it is difficult to tell the truth at GICs. Additionally, reliable psychiatric diagnoses can be made in GICs.

In our study, the patients mostly had major regret (all seven female-assigned at birth patients) and two male-assigned at birth patients had minor regret. Even the patients who were non-binary regretted having started the GAT. The suffering and long-lasting effects on their lives continued after stopping the treatment. In this study, all the patients except one needed medical treatment: surgery (67%), hormones (67%), epilation (67%), phoniatric services (33%), and psychophysical physiotherapy (22%). The majority (78%) of the detransitioners were referred to psychiatric services.

The reasons why people transitioned in the first place were quite similar in our study sample in comparison with previous studies. There were no external reasons that made patients in our study detransition. No one talked about discrimination. Instead, misogyny was mentioned often and becoming a woman was reported to be hard. Misogyny was not only encountered by the FtM subgroup, but also by the MtF group. Most of the study participants thought that their gender dysphoria was caused by trauma or a mental health condition (autism spectrum or dissociative disorder). One patient thought they had confused being gay with being transgender.

### Changes to the Helsinki University Hospital Gender Identity Clinic’s Process

Based on the results of this study and the requests from the detransitioners (Table 4), we made changes in the HUS GIC. First, referrals are not required when returning to the GIC with detransition wishes (“Make it easier to get in contact”). In Finland, transgender patients are treated through special services that GICs supervise according to the law. An adequate referral is needed to access the GIC, as with any specialized elective outpatient clinic. Among detransitioners, the threshold to seek help may be high. Therefore, we let them re-access our services without delay, not requiring a formal referral. Second, we added closer cooperation with the psychiatric staff that serves the patients by including an appointment with the GIC, the psychiatric staff and a patient (“I want the GIC to get in touch with my psychiatrist”). In addition, we preferably accept referrals from the patient’s psychiatrist if the patient has one. Third, cognitively oriented brief therapies are available for all our patients free of charge (“Take time to discuss”; “Recommend psychotherapy to me”). Fourth, we educate our staff to concentrate on emphasizing professional neutrality and empathy without premature expectations and over-involvement. Shockingly, in our sample (as seen elsewhere, “having been too enthusiastically affirmed” (Exposito-Campos, 2021)) some patients felt that the staff of the GICs were trying to convince patients they were trans. There have not been official appeals on the subject, so it is difficult to investigate these two claims officially. However, we take it very seriously and further encourage professional neutrality in the evaluation process. Remaining sensitive, open, and understanding while maintaining neutrality and safe structures may be a life-long lesson to learn. Due to the Finnish Trans Law, our evaluation process is multi-professional and thorough. Detransitioners wished that they would have been evaluated even more thoroughly, with an emphasis on dissociative disorders, trauma, and neuropsychiatric conditions that had remained undiagnosed or underestimated. All patients had childhood traumas that they found to be significant, but only one had PTSD diagnoses. Finally, a greater focus on childhood and childhood families has been added to the evaluation process.

Psychological assessment remains an important part of the gender identity evaluation; of the nine study participants, the psychologist had initially expressed concerns about the psychiatric well-being of seven. The systematic evaluation of attachment patterns might be useful. If a patient has a trauma background, psychotherapy might be necessary.

Even though most adults seeking GAT benefit from it and are satisfied with the treatment, it is important to acknowledge, support and evaluate those regretting treatments and/or who wish to detransition, and to learn from them. At minimum, the personal suffering of our patients demands that. Those who detransition have a high amount of childhood and sexual trauma, eating disorder symptoms, borderline personality disorders and psychotic symptoms. Evaluating and treating serious psychiatric illnesses first, to determine if the patients’ dysphoria resolves without GAT, might reduce the cases of detransitioning. Sufficient psychotherapy might help prior to irreversible GAT. The need for more research is urgent, and a wider, unprejudiced voice in public discussion about detransitioning and regret is needed. It is important to encourage detransitioners to notify the GIC that they detransitioned, as it would provide valuable information to clinicians about patient outcomes.

The results of this study should be used to inform the evaluation process, counseling, informed consent, and medical decision-making for patients with gender dysphoria. The results do not support eliminating transition services nor do they support proceeding to transition without adequate evaluation (MacKinnon et al., 2023).

### Strengths and Limitations

To our knowledge, this is one of the first systematic studies of the psychiatric assessment results and subjective experiences of a sample of patients who detransitioned after any GAT. Assembling the study group from the clinic’s patients and meeting them in person leads to a smaller patient group but adds important knowledge and prevents common problems with sample bias. The selected study sample was naturalistic in that all the study participants sought treatment from the GIC on their own initiative, with aims to detransition. The limitations of this study are the small size of the naturalistic clinical sample and the likelihood that the patients who returned to the GIC for detransition represent only a minority of patients who detransitioned. There was also no control group for the detransitioners.
